# Identification of Genes Preferentially Expressed in Stomatal Guard Cells of *Arabidopsis thaliana* and Involvement of the Aluminum-Activated Malate Transporter 6 Vacuolar Malate Channel in Stomatal Opening

**DOI:** 10.3389/fpls.2021.744991

**Published:** 2021-10-08

**Authors:** Wenxiu Ye, Shota Koya, Yuki Hayashi, Huimin Jiang, Takaya Oishi, Kyohei Kato, Kohei Fukatsu, Toshinori Kinoshita

**Affiliations:** ^1^School of Agriculture and Biology, Shanghai Jiao Tong University, Shanghai, China; ^2^Institute of Transformative Bio-Molecule, Nagoya University, Nagoya, Japan; ^3^Graduate School of Science, Nagoya University, Nagoya, Japan

**Keywords:** *ALMT6*, *Arabidopsis*, blue light, malate, proton pump, stomatal opening

## Abstract

Stomatal guard cells (GCs) are highly specialized cells that respond to various stimuli, such as blue light (BL) and abscisic acid, for the regulation of stomatal aperture. Many signaling components that are involved in the stomatal movement are preferentially expressed in GCs. In this study, we identified four new such genes in addition to an aluminum-activated malate transporter, *ALMT6*, and GDSL lipase, *Occlusion of Stomatal Pore 1* (*OSP1*), based on the expression analysis using public resources, reverse transcription PCR, and promoter-driven β-glucuronidase assays. Some null mutants of GC-specific genes evidenced altered stomatal movement. We further investigated the role played by ALMT6, a vacuolar malate channel, in stomatal opening. Epidermal strips from an *ALMT6-*null mutant exhibited defective stomatal opening induced by BL and fusicoccin, a strong plasma membrane H^+^-ATPase activator. The deficiency was enhanced when the assay buffer [Cl^–^] was low, suggesting that malate and/or Cl^–^ facilitate efficient opening. The results indicate that the GC-specific genes are frequently involved in stomatal movement. Further detailed analyses of the hitherto uncharacterized GC-specific genes will provide new insights into stomatal regulation.

## Introduction

Stomata that are formed by pairs of guard cells (GCs) in the shoot epidermis of plants are key regulators of gas exchange, such as CO_2_ uptake for photosynthesis and water loss during transpiration (Shimazaki et al., 2007; Munemasa et al., 2015). GCs respond to internal and external signals, such as light, CO_2_, phytohormones, and microbial elicitors, where the stomata remain either open or close (Murata et al., 2015; Inoue and Kinoshita, 2017). Many critical signaling components that are involved in GC signaling are preferentially expressed in GCs, such as Open Stomata 1 (OST1) (Mustilli et al., 2002), slow anion channel-associated 1 (SLAC1) (Negi et al., 2008; Vahisalu et al., 2008), high leaf temperature 1 (HT1) (Hashimoto et al., 2006), and aluminum-activated malate transporter 12 (ALMT12) (Meyer et al., 2010; Sasaki et al., 2010), suggesting that GC-specific genes are important candidates in hunting for new GC signaling components.

Blue light (BL) and red light (RL) are major cues for stomatal opening (Shimazaki et al., 2007; Inoue and Kinoshita, 2017). On BL perception, phototropins undergo autophosphorylation, which triggers signaling by BLUS1, BHP1, type 1 protein phosphatase (PP1), and its regulatory subunit PRSL1, in turn leading to phosphorylation of the penultimate threonine (penThr) residues of the plasma membrane (PM) H^+^-ATPases, and the subsequent binding of 14-3-3 proteins activates the H^+^-ATPases. More recently, RL was shown to induce the activation of GC PM H^+^-ATPases by phosphorylation (Ando and Kinoshita, 2018). PM H^+^-ATPases are important in terms of stomatal movement; the activation induces PM hyperpolarization, triggering a K^+^ influx through inward-rectifying K^+^ channels (Shimazaki et al., 2007; Inoue and Kinoshita, 2017). Together with the accumulation of K^+^, the increase of counter anions, such as malate, biosynthesized in GCs and/or apoplast and Cl^–^ from apoplast, and other osmolytes, such as sucrose, lower the water potential in GCs, leading to an inflow of water, the swelling of GCs, and eventually the stomatal opening (Shimazaki et al., 2007; Santelia and Lawson, 2016). Recently, it has been shown that the activation of PM H^+^-ATPases occurs upstream of starch degradation associated with BL-induced stomatal opening; this, combined with CO_2_ fixation in GC chloroplasts, yields the carbon skeletons required for malate synthesis (Horrer et al., 2016). Vacuoles accumulate most of the ions and water that control stomatal movement (Barbier-Brygoo et al., 2011). The electrophysiological experiments revealed that the ALMT6 and ALMT9 vacuole channels facilitated malate and Cl^–^ import (Meyer et al., 2011); ALMT9 played a critical role in the light-induced stomatal opening (De Angeli et al., 2013).

To identify new signaling components involved in the light-induced stomatal opening, we reasoned that GC preferentially expressed genes are good candidates and identified four new such genes by the analyses of public resources, reverse transcription PCR (RT-PCR), and promoter GUS assay in *Arabidopsis thaliana*. Functional analysis revealed that some of the GC-specific genes in addition to ALMT6 are critical in the light-induced stomatal opening.

## Materials and Methods

### Plant Materials and Growth Conditions

All *A. thaliana* strains were grown in soil under a photon flux density of 50 μmol m^–2^ s^–1^ and a 16-h-light/8-h-dark regime. The temperature and the relative humidity were 23 ± 2°C and 55–70%, respectively. All mutants [*at5g18430* (SALK_116756), *almt6-1* (GABI_259D05; Meyer et al., 2011), *at1g33811* (GABI_492D11), *osp1-1* (SALK _106116; Tang et al., 2020), and *at3g23840* (GABI_180G04)] are in the Columbia ecotype background (Col-0).

### Isolation of Guard Cell Protoplasts and Mesophyll Cell Protoplasts

Guard cell protoplasts (GCPs) and mesophyll cell protoplasts (MCPs) were isolated from *glabra1-1* (*gl1*) as described previously (Okumura et al., 2016).

### Reverse Transcription PCR

RNAs from *gl1* GCPs, MCPs, rosette leaves, roots, petioles, stems, flowers, and etiolated seedlings were extracted using the RNeasy Plant Mini Kit (QIAGEN) according to the protocol of the manufacturer. Complementary DNA was synthesized using the PrimeScript II 1st strand cDNA Synthesis Kit (Takara). The PCR primers are listed in Supplementary Table 1.

### Promoter GUS Assay

The promoter regions (3-kb upstream of the start codons) of *AT5G18430*, *ALMT6*, *AT1G33811*, *OSP1*, *AT3G23840*, and *AT3G17070* were amplified in two PCR steps using the primers listed in Supplementary Table 2 and cloned into pCR8/GW/TOPO followed by subcloning into pGWB433 binary vector. The vectors were transformed into *Agrobacterium* GV3101, which were then used to transform Col-0 by floral dip. Transformants were selected using kanamycin and carbenicillin and subjected to GUS staining at various developmental stages.

### Stomatal Aperture Measurement

The stomatal aperture measurement was performed as described previously (Tomiyama et al., 2014; Toh et al., 2018). Epidermal tissues and leaf disks were prepared from dark-adapted plants and subjected to light illumination and fusicoccin (FC) treatment. The apertures were measured under a microscope (Olympus).

### Immunohistochemical Staining of Plasma Membrane H^+^-ATPase in Guard Cells

The immunohistochemical staining was performed as described previously (Hayashi et al., 2011). Epidermal tissues were prepared from dark-adapted plants and subjected to light illumination and FC treatment. RL (50 μmol m^–2^ s^–1^) was illuminated for 20 min (Red), and BL (10 μmol m^–2^ s^–1^) was illuminated with superimposed on RL for 2.5 min (Red + Blue). FC at 10 μM was applied to the epidermal tissue for 5 min in the dark (FC). PM H^+^-ATPases and the phosphorylation level of the penThr were detected using specific antibodies against the catalytic domain of AHA2 (anti-PM H^+^-ATPase antibody) and phosphorylated Thr-947 in AHA2 (anti-pThr) (Hayashi et al., 2010).

### Accession Numbers

Sequence data can be found in the Arabidopsis genome database TAIR10 under the following accession numbers: ALMT6 (AT2G17470), OSP1 (AT2G04570), ALMT9 (AT3G18440), AT1G02980, AT1G12030, AT1G33811, AT2G32830, AT3G17070, AT3G23840, and AT5G18430.

## Results

### Genes Preferentially Expressed in *Arabidopsis* Guard Cells

**FIGURE 1:**
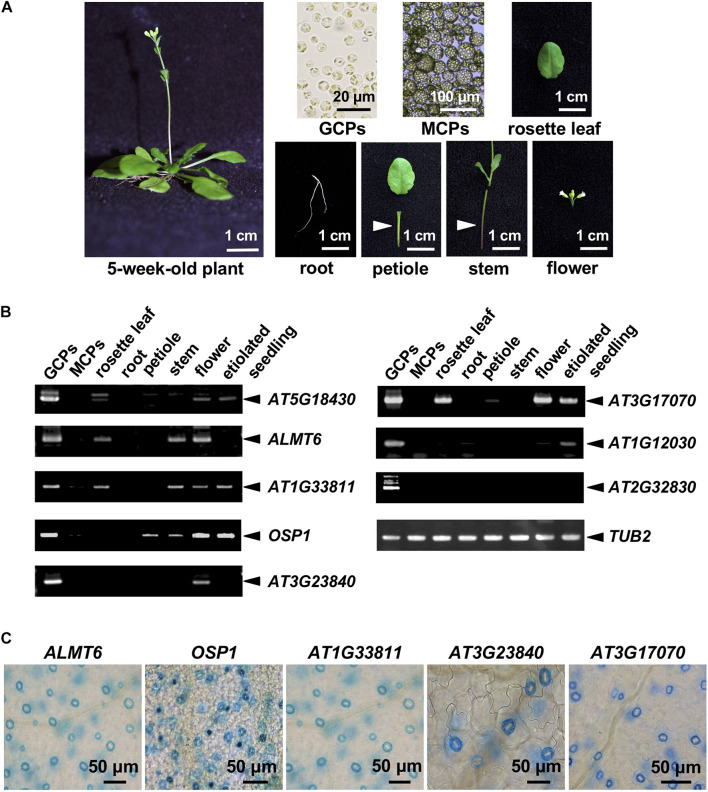
Guard cell (GC)-preferentially expressed genes. **(A)** The plant organs, tissues, and cells used in the analysis. **(B)** GC-preferentially expressed genes as revealed by RT-PCR using the materials shown in panel **(A)**. **(C)** GC-preferentially expressed genes in GUS-reporter-bearing plants.

We analyzed publicly available microarray data on GCPs and MCPs (Yang et al., 2008), and those of the *Arabidopsis* eFP browsers^1^. The inclusion criteria were as follows: (1) a microarray GCP signal unique to GCPs or at least fourfold higher than the MCP signal and (2) the “Tissue-Specific” criteria of the *Arabidopsis* eFP browsers indicated GC-specific expression. We retrieved 124 candidate genes and checked their expression levels by RT-PCR in various cells, tissues, and organs of *A. thaliana* (Figure 1A). A total of 10 genes were strongly expressed in GCPs but not in MCPs and roots; these included *SLAC1*, the cation/H^+^ exchanger-encoding *AtCHX20*, and genes encoding the GDSL lipases *OSP1* and *ALMT6* (which are preferentially expressed in GCs; Padmanaban et al., 2007; Negi et al., 2008; Vahisalu et al., 2008; Tang et al., 2020) and six functionally uncharacterized genes. The RT-PCR data for *OSP1*, *ALMT6*, and the uncharacterized genes are shown in Figure 1B.

**FIGURE 2:**
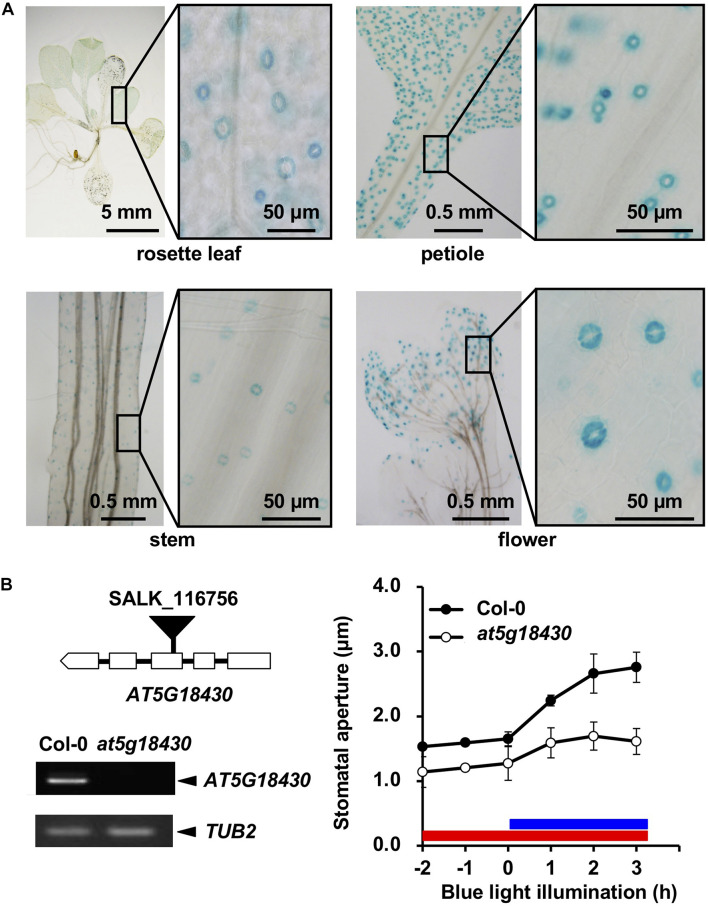
Expression of *At5g18430* and the stomatal phenotype of the *at5g18430* mutant. **(A)** Expression pattern of *At5g18430* in GUS-reporter-bearing plants. **(B)** The stomatal phenotype of the *at5g18430* mutant. Left: A schematic of the T-DNA insertion site in *at5g18430* and the transcript levels in leaves. Right: blue light (BL)-induced stomatal opening in Col-0 and *at5g18430* epidermal tissues. The red bar indicates the red light (RL) illumination period (50 μmol m^–2^ s^–1^). The blue bar indicates the BL illumination period (10 μmol m^–2^ s^–1^). Epidermal tissues were incubated in 50 mM KCl, 0.1 mM CaCl_2_, and 10 mM Mes-BTP (pH 6.5). Averages from three independent experiments are shown. Error bars: SDs (*n* = 3).

**FIGURE 3:**
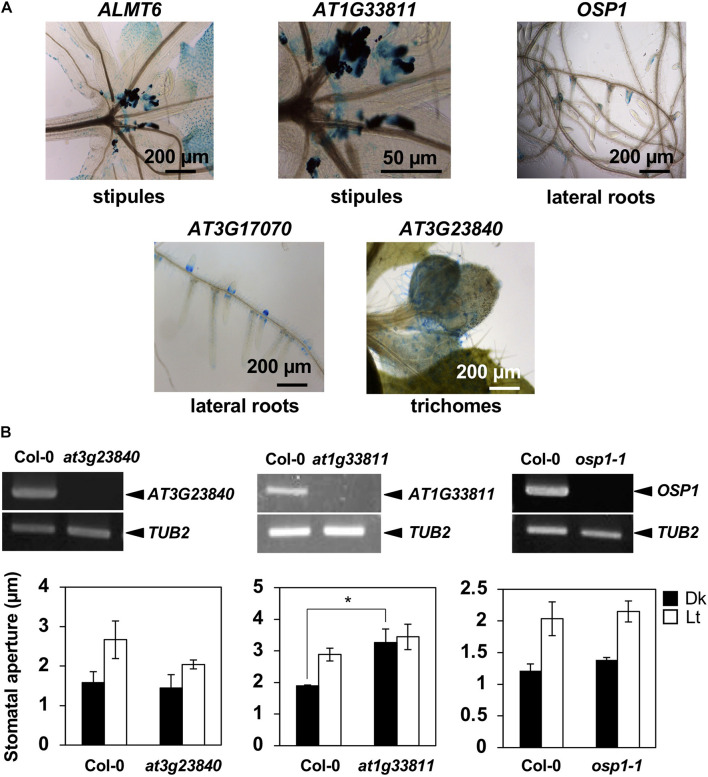
Expression patterns of GC-preferentially expressed genes and the stomatal phenotypes of knockout mutants. **(A)** Gene expression in tissues other than stomata as revealed by the GUS-reporter assay. **(B)** Upper panels show the RT-PCR of target genes. Lower graphs show BL-induced stomatal opening in *at3g23840*, *at1g33811*, and *osp1-1* null mutants. Epidermal tissues (*at3g23840* and *at1g33811* mutants) or leaf disks (*osp1-1* mutant) were incubated in 50 mM KCl, 0.1 mM CaCl_2_, and 10 mM Mes-BTP (pH 6.5). Dark bars, dark treatment for 3 h (Dk); white bars, light treatment for 3 h (RL, 50 μmol m^–2^ s^–1^; BL, 10 μmol m^–2^ s^–1^) (Lt). Average values from three independent experiments are shown. Error bars: SDs (*n* = 3).

To further confirm preferential GC expression in intact plants, we constructed transgenic plants expressing the reporter β-GUS-encoding gene driven by promoter regions ranging to about 3-kb upstream of the start codons. *pALMT6*:*GUS* and *pOSP1*:*GUS* exhibited the high-level GUS activity in GCs (in particular) (Figure 1C), consistent with previous findings (Meyer et al., 2011; Tang et al., 2020). Also, the *AT1G33811*, *AT3G23840*, *AT3G17070*, and *AT5G18430* promoters drove GC-preferential GUS expression (Figures 1C, 2A). The *ALMT6*, *AT1G33811*, *OSP1*, *AT3G17070*, and *AT3G23840* promoters drove GUS expression in stipules, lateral roots, and trichomes (Figure 3A); however, the *AT5G18430* promoter was active in GCs only (Figure 2A). The *AT1G12030* and *AT2G32830* promoters never drove GUS expression, rendering the analyses difficult. This is probably due to the lack of or weak activity of the 3-kb upstream promoter regions of these two genes.

### Stomatal Phenotypes of Null Mutants of Genes Preferentially Expressed in Guard Cells

We prepared null mutants of *OSP1*, *AT1G33811*, *AT3G23840*, *AT3G17070*, and *AT5G18430*. Although we failed to obtain the knockout mutants of *at3g17070* (SALK_121694), the null mutants of *at5g18430* (SALK_116756) and *at3g23840* (GABI_180G04) were impaired in BL-induced stomatal opening (Figure 2B) and light-induced stomatal opening (Figure 3B), respectively. Interestingly, the stomata of *at1g33811* (GABI_492D11) null mutant were open even in the dark (Figure 3B). The null mutant of *OSP1*, i.e., *osp1-1*, exhibited a normal stable-status stomatal opening in the light (Figure 3B and Supplementary Figure 1), which is consistent with a previous report (Tang et al., 2020). Thus, the previously uncharacterized GC-specific genes *AT1G33811*, *AT3G23840*, and *AT5G18430* may be involved in stomatal movement.

### Characterization of the *ALMT6-*Null Mutant in Terms of Blue Light-Induced Stomatal Opening

**FIGURE 4:**
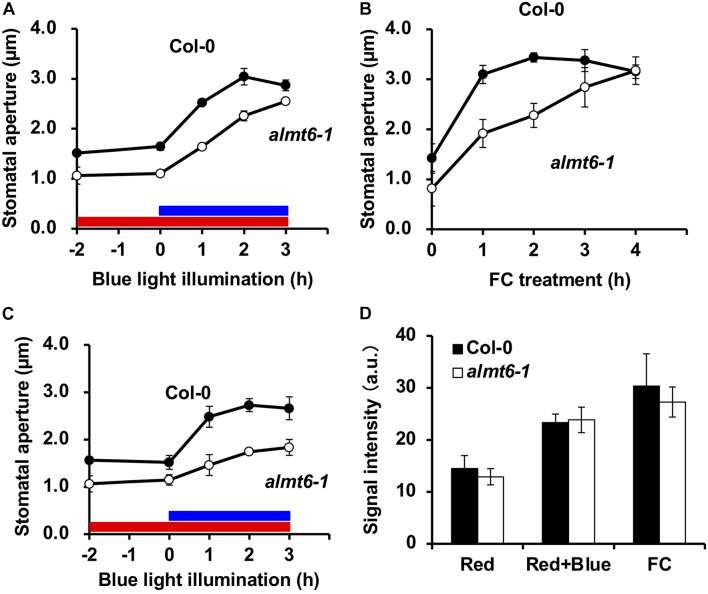
Stomatal phenotypes in wild-type Col-0 and *almt6-1* mutant. **(A)** BL-induced stomatal opening in wild-type Col-0 and *almt6-1* mutant epidermal tissues. Red bar: RL illumination period (50 μmol m^–2^ s^–1^). Blue bar: BL illumination period (10 μmol m^–2^ s^–1^). **(B)** Fusicoccin (FC)-induced stomatal opening in Col-0 and *almt6-1* epidermal tissues. In panels **(A,B)**, epidermal tissues were incubated in 50 mM KCl, 0.1 mM CaCl_2_, and 10 mM Mes-BTP (pH 6.5) and then subjected to light and 10 μM FC treatments. **(C)** BL-induced stomatal opening in low Cl^–^ (0.2 mM) buffer of Col-0 and *almt6-1* epidermal tissues. Epidermal tissues were incubated in 50 mM potassium gluconate, 0.1 mM CaCl_2_, and 10 mM Mes-BTP (pH 6.5). Averages from three independent experiments are shown. Error bars: SDs (*n* = 3). The other conditions are those in panel **(A)**. **(D)** Immunohistochemical detection of BL- and 10 μM FC-induced phosphorylation of PM H^+^-ATPase in Col-0 and *almt6-1* GCs. GC fluorescence was quantified using an anti-pThr antibody and Alexa Fluor 488-conjugated secondary antibody as described in section “Materials and Methods.” Bars: Averages from three independent experiments. Error bars: SDs (*n* = 3). a.u., arbitrary units.

The ALMT6 is a vacuolar malate channel (Meyer et al., 2011). We explored the stomatal movements of the *almt6-1* mutant in detail. The expression of *ALMT9* in GCs is not altered in *almt6-1* (Supplementary Figure 2). As shown in Figure 4A, the stomata of *almt6-1* were slightly narrower than wild type under the dark and RL condition, as well as opened but less efficiently to a similar size of those of wild type on BL illumination. Less efficiency of *almt6-1* stomatal opening in response to 10 μM FC, an activator of PM H^+^-ATPase, was more prominent compared with the case of BL-induced stomatal opening (Figure 4B). Stomatal apertures in the *almt6-1* mutant were comparable to those in wild type when epidermal peels were treated with light or FC for more than 3 or 4 h, respectively (Figures 4A,B). Thus, ALMT6 may be required for stomatal opening induced by BL and FC. It is worthy of note that usually BL-insensitive mutants, such as *phot1 phot2* double mutant, show completely insensitive phenotype to BL but open normally in response to FC (Kinoshita et al., 2001). The less efficient phenotype of stomatal opening in *almt6-1* is very similar to a *kincless* mutant (Lebaudy et al., 2008) and *aks1 aks2* mutant (Takahashi et al., 2013), which shows the low activity of PM inward K^+^ channels in GCs, suggesting that the deficient of ion transport for stomatal opening leads to less efficiency of stomatal opening.

The ALMT6 transports (principally) malate and fumarate but Cl**^–^** to a lesser extent (Meyer et al., 2011); we thus explored how Cl**^–^** affected BL-induced stomatal opening. The usual stomatal assay buffer contains 50 mM KCl, 0.1 mM CaCl_2_, and 10 mM Mes-BTP (pH 6.5). To exclude exogenous Cl**^–^**, we evaluated stomatal opening in a buffer with 50 mM potassium gluconate, 0.1 mM CaCl_2_, and 10 mM Mes-BTP (pH 6.5). Gluconate does not readily cross the PM. Figure 4C shows that the 3-h BL-induced stomatal opening at a low [Cl**^–^**] was impaired in the *almt6-1* mutant in terms of both speed and amplitude.

### Blue Light- and FC-Induced Phosphorylation of Plasma Membrane H^+^-ATPase in the *almt6-1* Mutant

Both BL and FC induce the phosphorylation of the penThr of PM H^+^-ATPases, in GCs, which provides a driving force for stomatal opening (Inoue and Kinoshita, 2017). Thus, we immunohistochemically investigated the effects of BL and FC on the phosphorylation status of GC PM H^+^-ATPase; such phosphorylation was not impaired in the *almt6-1* mutant (Figure 4D). The amount of PM H^+^-ATPase in *almt6-1* under BL and FC was comparative to that in wild type (Supplementary Figure 3). The *almt6* mutation did not affect PM H^+^-ATPase phosphorylation and amount in response to BL and FC.

## Discussion

In this study, we identified 10 genes including *SLAC1*, Cation/H^+^ Exchanger *AtCHX20*, GDSL lipases *OSP1* and *ALMT6*, preferentially expressed in GCs based on the analyses of public resources, RT-PCR, and promoter GUS assay (Figures 1–3). Of these, *AT1G33811*, *AT3G17070*, *AT3G23840*, and *AT5G18430* have not been functionally characterized in stomata. Among these four genes, three genes, namely, *AT1G33811*, *AT3G23840*, and *AT5G18430*, were found involved in the stomatal movement (Figures 2, 3). Remarkably, two of them, *AT1G33811* and *AT5G18430*, are members of the GDSL family of serine esterases/lipases (Akoh et al., 2004), indicating the importance of this family in regulating stomatal movement. Tang et al. (2020) found that a GDSL lipase, i.e., OSP1, is preferentially expressed in GCs, and *osp1* mutants showed low stomatal conductance and high leaf temperature due to a high percentage of occluded stomata. Detail biological and/or biochemical analyses revealed that OSP1 is required for wax biosynthesis and proper formation of the stomatal outer cuticular ledge (Tang et al., 2020). Interestingly, *osp1* mutants were also impaired in abscisic acid (ABA)-induced stomatal closure, indicating a potential role of OSP1 in stomatal movement (Tang et al., 2020). It would be very interesting to investigate whether AT1G33811 and AT5G18430 have similar functions as OSP1. *AT3G23840*, previously named as *CER26-like*, is probably related to very long-chain fatty acid metabolism (Pascal et al., 2013), and a knockout mutant, *at3g23840*, showed reduced light-induced stomatal opening (Figure 3B). Future study is needed to provide a detailed mechanism mediated by those genes in stomatal movement.

The detailed analyses of expression pattern by GUS-reporter assay of *ALMT6*, *OSP1*, *AT1G33811*, *AT3G17070*, *AT3G23840*, and *AT5G18430* revealed that promoter regions, i.e., 3-kb upstream of the start codon, drive GC-preferential gene expression with *AT5G18430* promoter showing the most GC-selective property (Figures 1–3). The 1-kb upstream region of the *AT3G23840* start codon was promoter-active in flowers (Pascal et al., 2013). The 3-kb promoter region of *AT3G23840* studied here showed strong GC signals in addition to flower (Figure 1B). The 3-kb promoter region of *ALMT6* studied here was more GC-specific than a 1.8-kb region used previously, which exhibited strong activities in floral tissues, such as sepals, petals, and anthers (i.e., not only GCs; Meyer et al., 2011) (Figures 1C, 3A). These results suggest that specific elements and their patterns determine the GC-specific activity of promoters. So far, several GC-preferentially expressed genes, such as *GC1* (Yang et al., 2008) and *MYB60* (Cominelli et al., 2005), were reported. However, these genes were out of our strict criteria, indicating that the genes reported in this study are more specific in GCs (Supplementary Figure 4). Cell type-specific promoters are important tools to study gene function and of great application potential (Imlau et al., 1999; Yang et al., 2008; Wang et al., 2014). The promoters identified in this study thus add the options for promoter engineering for GC-specific gene regulation.

Both ALMT6 and ALMT9 were initially identified as channels mediating malate accumulation in vacuoles (Kovermann et al., 2007; Meyer et al., 2011). Later, ALMT9 was shown to be a Cl**^–^** channel regulated by cytosol malate and to be required for the light-induced stomatal opening (De Angeli et al., 2013). As shown in Figures 4A,B, *almt6-1* mutant opened stomata with less efficiency in response to BL illumination and FC treatment, suggesting that ALMT6 is also required for BL- and FC-induced stomatal opening. Notably, *almt6-1* mutant showed significant impairment in BL-induced stomatal opening under low Cl^–^ condition (Figure 4C), suggesting that ALMT6, such as ALMT9, also contributes Cl^–^ influx to vacuole during stomatal opening. Since both *almt6* and *almt9* single mutants are impaired in the light-induced stomatal opening and there is no compensation of *ALMT9* expression in *almt6* (Supplementary Figure 2), it is possible that ALMT6 and ALMT9 function in an additive and/or cooperative manner. In the cooperative mode, a bold hypothesis is that ALMT6 and ALMT9 form heteromeric channels mediating anion accumulation in GC vacuoles as they were shown to form tetramer channels in heterosystems (Zhang et al., 2013). Future electrophysiological and genetic studies such as phenotyping using *almt6 almt9* double mutant are needed to clarify the contribution of ALMT6 and ALMT9 in stomatal opening.

In this study, we identified preferentially expressed genes in GCs and found that some uncharacterized genes are involved in stomatal movement. Especially, to our knowledge, AT5G18430 shows the most specific expression in GCs. In addition, we showed evidence that ALMT6 is important for BL- and FC-induced stomatal opening. Further detailed analyses of the uncharacterized GC-specific genes will provide novel understandings for stomatal movement.

## Data Availability Statement

The original contributions presented in the study are included in the article/Supplementary Material, further inquiries can be directed to the corresponding author/s.

## Author Contributions

WY, SK, and TK designed the experiments. WY, SK, YH, HJ, TO, KK, and TK performed the experiments. WY, SK, YH, and TK wrote the manuscript. All authors contributed to the article and approved the submitted version.

## Conflict of Interest

The authors declare that the research was conducted in the absence of any commercial or financial relationships that could be construed as a potential conflict of interest.

## Publisher’s Note

All claims expressed in this article are solely those of the authors and do not necessarily represent those of their affiliated organizations, or those of the publisher, the editors and the reviewers. Any product that may be evaluated in this article, or claim that may be made by its manufacturer, is not guaranteed or endorsed by the publisher.

## Footnotes

1
https://bar.utoronto.ca/efp/cgi-bin/efpWeb.cgi
