# Supplementary material for: Identification of Genes Preferentially Expressed in Stomatal Guard Cells of Arabidopsis thaliana and Involvement of the Aluminum-Activated Malate Transporter 6 Vacuolar Malate Channel in Stomatal Opening
Source: Front Plant Sci. 2021 Oct 8;12:744991. doi: 10.3389/fpls.2021.744991 (PMC8531587; doi:10.3389/fpls.2021.744991)
Supplement: Supplementary file 1 [file Data_Sheet_1.pdf]

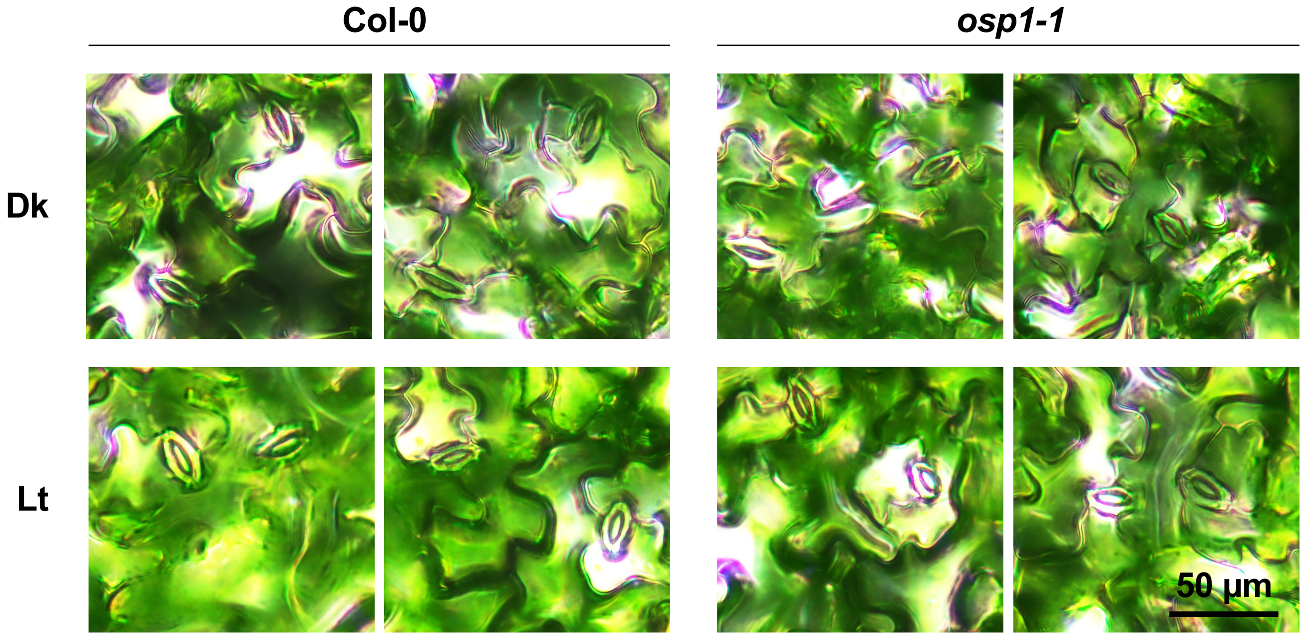

SUPPLEMENTARY FIGURE 1. Pictures of typical stomata from Col-0 and *osp1-1* mutant in leaf discs. Dk, dark treatment for 3 hr; Lt, light treatment for 3 hr (RL, 50 µmol m^{-2} s^{-1}; BL, 10 µmol m^{-2} s^{-1}). The other conditions are those in **Figure 3B**.
