# Supplementary material for: Identification of Genes Preferentially Expressed in Stomatal Guard Cells of Arabidopsis thaliana and Involvement of the Aluminum-Activated Malate Transporter 6 Vacuolar Malate Channel in Stomatal Opening
Source: Front Plant Sci. 2021 Oct 8;12:744991. doi: 10.3389/fpls.2021.744991 (PMC8531587; doi:10.3389/fpls.2021.744991)
Supplement: Supplementary file 2 [file Data_Sheet_2.pdf]

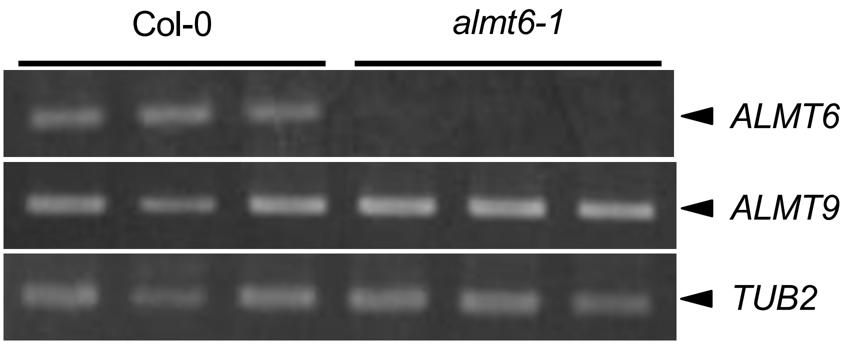

SUPPLEMENTARY FIGURE 2. Transcript levels of *ALMT6* and *ALMT9* in guard cell-enriched epidermal strips of Col-0 and *almt6-1*. Tubulin served as the control. Results of three independent experiments were shown.
