# Supplementary material for: Identification of Genes Preferentially Expressed in Stomatal Guard Cells of Arabidopsis thaliana and Involvement of the Aluminum-Activated Malate Transporter 6 Vacuolar Malate Channel in Stomatal Opening
Source: Front Plant Sci. 2021 Oct 8;12:744991. doi: 10.3389/fpls.2021.744991 (PMC8531587; doi:10.3389/fpls.2021.744991)
Supplement: Supplementary file 3 [file Data_Sheet_3.pdf]

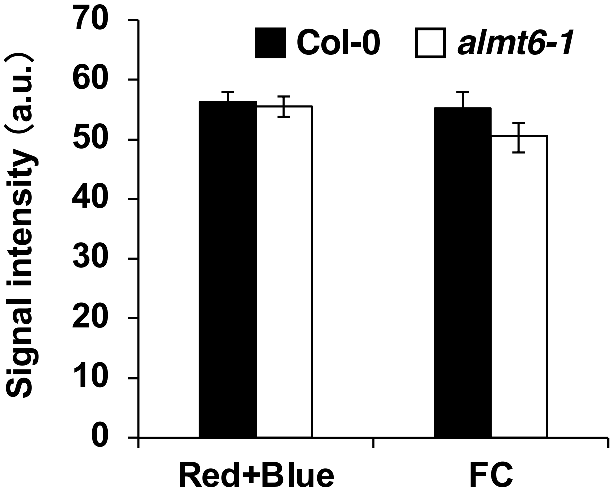

SUPPLEMENTARY FIGURE 3. Immunohistochemical detection of amount of PM H^+-ATPase in Col-0 and *almt6-1* guard cells. GC fluorescence was quantified using an anti-PM H^+-ATPase antibody and Alexa Fluor 488-conjugated secondary antibody as described in the Materials and Methods. Bars: Averages from three independent experiments. Error bars: SDs (n=3). a.u., arbitrary units.
