# Supplementary material for: Identification of Genes Preferentially Expressed in Stomatal Guard Cells of Arabidopsis thaliana and Involvement of the Aluminum-Activated Malate Transporter 6 Vacuolar Malate Channel in Stomatal Opening
Source: Front Plant Sci. 2021 Oct 8;12:744991. doi: 10.3389/fpls.2021.744991 (PMC8531587; doi:10.3389/fpls.2021.744991)
Supplement: Supplementary file 4 [file Data_Sheet_4.pdf]

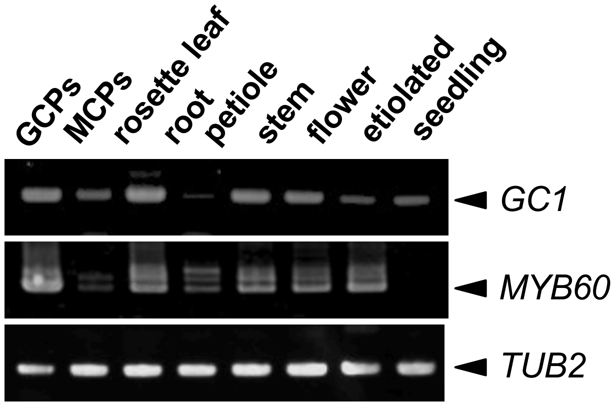

SUPPLEMENTARY FIGURE 4. Expression patterns of known GC preferentially expressed genes. RT-PCR of *GC1* and *MYB60* using materials shown in **Figure 1A**. Primers were same as previous reports (Yang et al., 2008; Cominelli et al., 2005).
