# Supplementary material for: Identification of Genes Preferentially Expressed in Stomatal Guard Cells of Arabidopsis thaliana and Involvement of the Aluminum-Activated Malate Transporter 6 Vacuolar Malate Channel in Stomatal Opening
Source: Front Plant Sci. 2021 Oct 8;12:744991. doi: 10.3389/fpls.2021.744991 (PMC8531587; doi:10.3389/fpls.2021.744991)
Supplement: Supplementary file 5 [file Data_Sheet_5.PDF]

Supplementary Table 1. Primers for RT-PCR

Primer names	Sequences (5'-3')
AT1G02980 F	GATGAGCCACCATTTGACCC
AT1G02980 R	GTTTGATAAGGGCACTTCCCTC
AT1G12030 F	TGTGAAGGAGAGGTTACGGG
AT1G12030 R	CTFTTTCAGCTCCTCAGGTG
AT1G33811 F	TTGCTTTCTCTTGCGAGGGC
AT1G33811 R	ATACTTGGTTCGGTCAGGGC
OSP1 F	TTGCTTCTGCTGCCACTGGC
OSP1 R	CATCTCGAACATCCCTGTCGC
ALMT6 F	CATCCTTACTGTCGTCGTGG
ALMT6 R	CTGTAAGGTCCATGTGGAGG
AT2G32830 F	TAATGGTGTGGCCTTCTGCG
AT2G32830 R	GGTTGCTGCTGTAATAGGCG
AT3G17070 F	ATCCTACTTCTCCTGCAGCC
AT3G17070 R	TGGGGACAAATGTGGCTTCC
AT3G23840 F	TATCAACCGTGAGTGCGTCC
AT3G23840 R	ATCTCGAATTCGCCGTCTCC
AT5G18430 F	GACGTGCTTTTTTCGTGTTCGG
AT5G18430 R	GACGTATAACTCACGGTTCGG
ALMT9 F	GAATTCACCATCGGAGCAAC
ALMT9 R	GTAAGCAAGAAAACCCGGAAC
TUB2 F	CTGTCTCCAAGGGTTCCAGG
TUB2 R	GGTTCAAATCACCAAAGCTGGG
