# Supplementary material for: Identification of Genes Preferentially Expressed in Stomatal Guard Cells of Arabidopsis thaliana and Involvement of the Aluminum-Activated Malate Transporter 6 Vacuolar Malate Channel in Stomatal Opening
Source: Front Plant Sci. 2021 Oct 8;12:744991. doi: 10.3389/fpls.2021.744991 (PMC8531587; doi:10.3389/fpls.2021.744991)
Supplement: Supplementary file 6 [file Data_Sheet_6.PDF]

Supplementary Table 2. Primers for amplification of promoter regions

Primer names	Sequences (5'-3')
pALMT6 1st F	CAGATCACTTATTTCTTCTGGATATGGG
pALMT6 2nd F	TTGCAGTTTAGGAGTATTGTGTCTTTGCAG
pALMT6 R	TTTTTCCCCTAAATTACTCTGAAGAGAACC
pAt1g33811 1st F	TGTTATCAAAAGCACCGTGG
pAt1g33811 2nd F	ACCAAAAAAGGAAGTCTCGC
pAt1g33811 R	TAAGTTATAATTTAGTGAAATAATTGTAATGG
pAt3g23840 1st F	GTGAGAAAAGAGGCAGTGAGAGG
pAt3g23840 2nd F	GTTTTGCTCAAACTAATCACCTGTGGC
pAt3g23840 R	GGCTATGAGACAAAAGATGGTAGAG
pAt5g18430 1st F	CTTGAAGGTGTATTTTGTTAGGCC
pAt5g18430 2nd F	TGAGATTTCAGTTTCATTATTGGCC
pAt5g18430 R	AAAAGCGTTTTGTTTAATGACTGTGAC
pOSP1 1st F	CAGAAGCATCCTCAAGAACAGG
pOSP1 2nd F	CAATTTTCCAACCGCTGCTGC
pOSP1 R	AGTGTTTGTTTTTGTGGTTATGATTGTG
pAt3g17070 1st F	GCGACAAAGTGATTAGCC
pAt3g17070 2nd F	ATGAACGGCTTGTTCTCC
pAt3g17070 R	AACTTTCTCTTTTGTTTGGAATTTTAGG
